# Event-free survival in patients with polycythemia vera treated with ropeginterferon alfa-2b versus best available treatment

**DOI:** 10.1038/s41375-023-02008-6

**Published:** 2023-08-26

**Authors:** Heinz Gisslinger, Christoph Klade, Pencho Georgiev, Dorota Krochmalczyk, Liana Gercheva-Kyuchukova, Miklos Egyed, Petr Dulicek, Arpad Illes, Halyna Pylypenko, Lylia Sivcheva, Jiří Mayer, Vera Yablokova, Kurt Krejcy, Victoria Empson, Hans C. Hasselbalch, Robert Kralovics, Jean-Jacques Kiladjian, the PROUD-PV Study Group, Heinz Gisslinger, Jean-Jacques Kiladjian

**Affiliations:** 1 Department of Internal Medicine I, Division of Hematology and Blood Coagulation, Medical University Vienna, Vienna, Austria; 2 AOP Health, Vienna, Austria; 3 Medical University of Plovdiv, Plovdiv, Bulgaria; 4 Teaching Unit of the Hematology Department, University Hospital in Krakow, Krakow, Poland; 5 Clinical Hematology Clinic, Multiprofile Hospital for Active Treatment “Sveta Marina”, Varna, Bulgaria; 6 Department of Internal Medicine II, Kaposi Mor County Teaching Hospital, Kaposvar, Hungary; 7 Department of Clinical Hematology, University Hospital Hradec Kralove, Hradec Kralove, Czech Republic; 8 Department of Hematology, Faculty of Medicine, University of Debrecen, Debrecen, Hungary; 9 Department of Hematology, Regional Treatment and Diagnostics Hematology Centre, Cherkasy Regional Oncology Centre, Cherkasy, Ukraine; 10 First Department of Internal Medicine, Multiprofile Hospital for Active Treatment - HristoBotev, Vratsa, Bulgaria; 11 Clinic of Internal Medicine - Hematology and Oncology, University Hospital Brno, Brno, Czech Republic; 12 Department of Hematology, Yaroslavl Regional Clinical Hospital, Yaroslavl, Russia; 13 Department of Hematology, Zealand University Hospital, Roskilde, University of Copenhagen, Copenhagen, Denmark; 14 Department of Laboratory Medicine, Medical University of Vienna, Vienna, Austria; 15 Université de Paris, AP-HP, Hôpital Saint-Louis, Centre d’Investigations Cliniques, INSERM, CIC1427 Paris, France

**Keywords:** Drug development, Myeloproliferative disease

## To the Editor:

Patients with polycythemia vera (PV), an incurable Philadelphia-negative myeloproliferative neoplasm, have shortened survival due to thrombohemorrhagic complications and evolution to myelofibrosis or acute myeloid leukemia [1, 2]. Clinical management of PV has primarily addressed the thrombotic risk, whereas prevention of disease progression remains a significant unmet need [3]. Interferon alfa is recognized as a disease-modifying treatment for PV that may reduce the risk of myelofibrotic progression and potentially prolong overall survival according to retrospective data, but prospective studies to confirm these findings have been lacking [4]. Final results from the large PROUD-PV trial and its extension CONTINUATION-PV are now available comparing ropeginterferon alfa-2b (BESREMi®) with hydroxyurea or best available treatment (BAT) over a total of 6 years.

PROUD-PV (#NCT01949805) was a phase three, randomized, controlled, open-label trial conducted in 48 centers in Europe enrolling patients diagnosed with PV (WHO 2008 criteria), who regardless of conventional risk status required cytoreduction, and were hydroxyurea-naïve or pre-treated for <3 years [5, 6]. Patients gave informed consent and were randomized 1:1 (stratified by age, thrombotic history, and hydroxyurea pretreatment) to receive ropeginterferon alfa-2b or control treatment (hydroxyurea). Dosing was escalated until blood counts normalized (ropeginterferon alfa-2b: from 50–100 µg to a maximum of 500 µg every two weeks; hydroxyurea: from 500 mg to 3000 mg daily). After one year, patients could roll over into CONTINUATION-PV (#NCT02218047) for 5 further years of treatment. Patients allocated to the control arm could switch to any standard cytoreductive therapy (i.e. BAT), those in the ropeginterferon alfa-2b arm could extend the dosing interval to three or four weeks. Detailed design and methodology have been published elsewhere [5, 6].

Efficacy outcomes over 6 years were analyzed in the full analysis set for CONTINUATION-PV; all safety data were evaluated regardless of inclusion in the extension study. Time to first risk event (thromboembolic events, disease progression or death [all causes]) was evaluated by Kaplan-Meier analyses and the groups compared by log-rank test and by Cox proportional hazards model. Complete hematologic response (CHR) defined according to modified European LeukemiaNet (ELN) criteria [5] was compared between the groups using a log-binomial regression model; rate ratio (RR) and 95% confidence intervals (CI) were calculated from estimates of regression coefficients. Discontinued patients were considered non-responders for CHR in the primary analysis; a sensitivity analysis utilizing imputation of the last observation carried forward (LOCF) was conducted. Comparison of molecular response defined by ELN criteria [7] between the arms employed the same method as for CHR. LOCF is reported for all molecular analyses. AEs were analyzed descriptively.

In PROUD-PV, 127 patients were treated in each arm of whom 95 in the ropeginterferon alfa-2b arm and 74 in the control arm rolled over into CONTINUATION-PV (Fig. S1). Baseline characteristics (at screening in PROUD-PV) in the extension study population were comparable between the study arms regarding age, time since diagnosis, *JAK2*V617F allele burden, non-driver mutations, spleen size, presence of PV-related symptoms and the history of thromboembolic events and cardiovascular risk factors.

Individualized dose-titration of ropeginterferon alfa-2b resulted in a median cumulative 4-weekly dose in the 6th year of treatment of 499 µg (IQR: ±268–782 µg), administered at an extended 3- or 4-week interval in most patients (61.9%). In the control arm, 87.8% of patients remained on hydroxyurea as of the 72-month assessment with a median dose of 1000 mg/day (IQR: 750–1500 mg). The study ended on 29 April, 2021 when all patients had been treated for ≥6 years; the maximum duration was 7.3 years. Cumulative exposure was 568 patient-years (ropeginterferon alfa-2b) and 451 patient-years (control treatment). A total of 67/95 patients in the ropeginterferon alfa-2b arm and 52/74 in the control arm completed the study, giving rise to equivalent discontinuation rates.

**Fig. 1:**
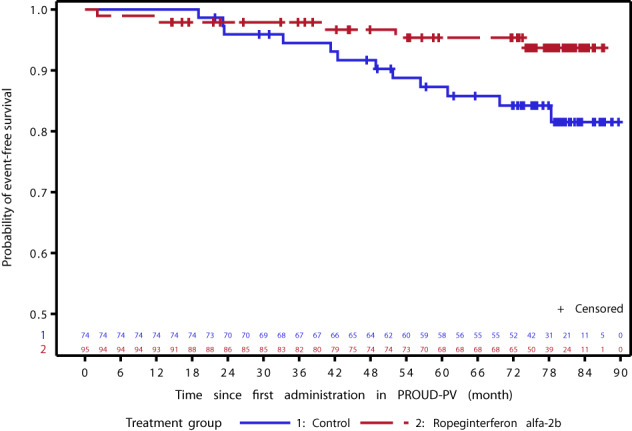
Probability of event-free survival in patients with PV in the ropeginterferon alfa-2b arm and control arm (CONTINUATION-PV full analysis set). Risk events were defined as thromboembolic events, disease progression or death.

Clinical events during PROUD-PV/CONTINUATION-PV were rare; median event-free survival was not reached (Fig. 1). The probability of event-free survival was significantly higher in the ropeginterferon alfa-2b arm compared with the control treatment group (0.94 versus 0.82; log-rank test; *p* = 0.04) as shown by analysis of the time to first risk event. The Cox proportional hazard ratio was 0.34 (95% CI: 0.12–0.97; *p* = 0.04). Risk events occurred in 5/95 patients (5.3%) in the ropeginterferon alfa-2b arm (first events: thromboembolic events [*n* = 2]; myelofibrosis [*n* = 1]; death [*n* = 2]) compared with 12/74 patients (16.2%) allocated to hydroxyurea/BAT (thrombotic events [*n* = 5]; myelofibrosis [*n* = 2]; acute leukemia [*n* = 2]; death [*n* = 3]).

These results align with potentially improved survival in interferon-treated PV patients compared with hydroxyurea treatment or phlebotomy in prior observational studies [4, 8]. Supporting a treatment effect in the current prospective study, risk factors associated with a poor prognosis in PV were balanced between the treatment arms at study start, largely due to stratification during randomization. Furthermore, no selection bias due to the roll-over was detected in analyses of patient characteristics and response rates comparing patients who enrolled or did not enroll into CONTINUATION-PV [5]. The final results of PROUD-PV/CONTINUATION-PV therefore indicate that the disease-modifying potential of ropeginterferon alfa-2b suggested by prior analyses [6] can now be quantified using hard clinical endpoints.

*JAK2*V617F allele burden is utilized as a surrogate endpoint for disease modification due to its central role in the pathophysiology of PV, and recent findings substantiate the notion that molecular response to treatment can alter the natural course of the disease [9].

Ropeginterferon alfa-2b profoundly diminished the *JAK2*V617F allele burden long-term: after 6 years, 62/94 patients (66.0%) achieved a molecular response (ELN criteria) compared with 14/72 (19.4%) in the control arm (risk ratio: 3.23 [2.01 to 5.19]; *p* < 0.0001). In line with our data published previously [6], median *JAK2*V617F allele burden at Month 72 was 8.5% versus 50.4% in the ropeginterferon alfa-2b and control arms, respectively (p < 0.0001) (Fig. S2). Moreover, allele burden >50%, which is presumed to reflect homozygosity for the *JAK2*V617F mutation and is associated with increased risks of thrombosis and disease progression [9–11], was found in only 11.6% of patients in the ropeginterferon alfa-2b arm in the 6th year of treatment compared with 50.0% of patients receiving hydroxyurea/BAT (*p* < 0.0001).

**Fig. 2:**
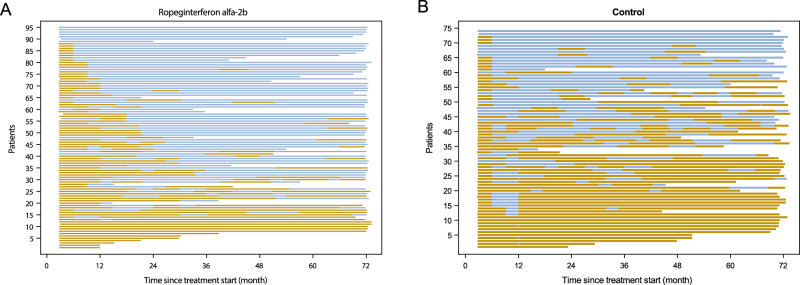
Time spent in complete hematologic response by treatment arm. Time spent in complete hematologic response based on ELN-defined targets for peripheral blood cell counts (hematocrit <45% without phlebotomy for ≥3 months, platelet count <400 × 109/L and leukocyte count <10 × 109/L) for individual patients with PV in the CONTINUATION-PV full analysis set (ropeginterferon alfa-2b arm: panel **A**; control arm: panel **B**). Patients are ordered by proportion of time in response (highest to lowest). Blue bars indicate a complete hematologic response at the latest available assessment; yellow bars indicate no response at the latest assessment.

Regarding hematologic parameters conventionally used to determine therapeutic efficacy, the significantly higher CHR rate in the ropeginterferon alfa-2b arm compared with the control arm reported at 3 years persisted after 6 years of treatment (month 72: 48/88 [54.5%] versus 22/63 [34.9%]; RR:1.55 [95% CI: 1.07 to 2.26; *p* = 0.02) [5]. A sensitivity analysis utilizing imputation of LOCF confirmed the higher response rate in the ropeginterferon alfa-2b arm (69/95 [72.6%] versus 35/74 [47.3%]; RR: 1.54 (95% CI: 1.18 to 2.00); *p* = 0.001) (Fig. S3). Despite the more gradual onset of CHR to ropeginterferon alfa-2b compared with control treatment (mainly hydroxyurea), patients treated with ropeginterferon alfa-2b spent a higher proportion of time in CHR than control treated patients (mean: 60.9% versus 41.2%, respectively; *p* = 0.04), underpinned by less fluctuation in responses according to ELN-defined targets for hematocrit, leukocyte count and platelet count (Figs. 2, S4).

While normalization of blood counts principally serves to minimize thrombotic risk in PV, leukocytosis has also been identified as a biomarker for disease evolution and overall survival [2, 12]. Longitudinal analysis of leukocytosis is essential, since a persistently elevated leukocyte trajectory is significantly associated with increased risk of disease progression (*p* = 0.0002) [12]. Patients receiving ropeginterferon alfa-2b in the current studies not only achieved a greater reduction in leukocyte count compared to baseline versus control (mean absolute changes: −6.67 × 10^9^/L versus −3.59 × 10^9^/L respectively [*p* < 0.0001] at Month 72), but spent significantly more time with normal leukocyte counts compared with hydroxyurea/BAT treated patients (mean proportion of time in response: 93.7% vs 80.9%; *p* = 0.02).

Final safety data from up to 7.3 years’ treatment in PROUD-PV and CONTINUATION-PV confirm the established positive safety profile of ropeginterferon alfa-2b [5, 6]. Only 14/127 (11.0%) of patients in the ropeginterferon alfa-2b arm and 3/127 (2.4%) in the hydroxyurea/BAT arm discontinued due to drug-related toxicity; the higher discontinuation rate in the ropeginterferon alfa-2b arm may reflect the intensive monitoring of certain interferon-alfa class effects, which mandated drug withdrawal according to the protocol. AEs were predominantly of mild or moderate severity, with comparable rates of grade ≥3 treatment-related AEs between the groups over the entire period (ropeginterferon alfa-2b: 15.7%; control: 16.5%). Furthermore, PV-associated AEs declined during ropeginterferon alfa-2b treatment, occurring in 7.1% of patients in the 6th year compared with 12.1% in the control arm.

Normal life expectancy may be achievable in PV based on comparable overall survival in interferon-treated patients and a matched US population (*p* = 0.3), contrasting with shortened survival (*p* = 0.03) in non-interferon treated patients at the same academic center [3]. Although overall survival was not evaluated, our findings lend support to this view, providing the first evidence that the durable hematologic and molecular responses observed with long-term ropeginterferon alfa-2b therapy are accompanied by improved event-free survival. This potential advantage should be considered when evaluating the individual risk-benefit relationship for ropeginterferon alfa-2b treatment in patients with PV.

### Supplementary information

Supplement

## Data Availability

The datasets generated during and/or analyzed during the current study are not publicly available to protect patient anonymity in this rare disease population but are available from the corresponding author on reasonable request.
