# Supplementary material for: Event-free survival in patients with polycythemia vera treated with ropeginterferon alfa-2b versus best available treatment
Source: Leukemia. 2023 Aug 26;37(10):2129–32. doi: 10.1038/s41375-023-02008-6 (PMC10539163; doi:10.1038/s41375-023-02008-6)

Supplementary material

This file includes supplementary results from the manuscript titled:

Event-Free Survival in Patients with Polycythemia Vera Treated with Ropeginterferon Alfa-2b versus Best Available Treatment

Members of the PROUD-PV Study Group

Principal Investigator	Affiliation
Heinz Gisslinger	Medical University Vienna, Department of Internal Medicine I, Clinical Division of Hematology and Hemostaseology, Vienna, Austria
Ella Willenbacher	Medical University Innsbruck, Department of Internal Medicine V (Hematology and Oncology), Innsbruck, Austria
Richard Greil	Salzburg Regional Hospital, University Department of Internal Medicine III, Salzburg, Austria
Ernst Forjan	Hanusch Hospital, Department of Internal Medicine III, Vienna, Austria
Veronika Buxhofer-Ausch	Internal Medicine I for Hematology with Stem Cell Transplantation, Hemostasis and Medical Oncology, Ordensklinikum Linz Elisabethinen, Linz, Austria
Franz Bauer	University Hospital Graz, University Clinic of Internal Medicine, Clinical Department of Hematology, Graz, Austria
Liana Gercheva-Kyuchukova	Multiprofile Hospital for Active Treatment "Sveta Marina", Varna, Bulgaria
Georgi Mihaylov	Specialized Hospital for Active Treatment of Hematological Diseases, Clinic of Clinical Hematology, Sofia, Bulgaria
Vera Stoeva	Specialized Hospital for Active Treatment of Hematological Diseases, Clinic of Clinical Hematology, Sofia, Bulgaria
Pencho Georgiev	University Multiprofile Hospital for Active Treatment "Sveti Georgi", Clinic of Hematology, Plovdiv, Bulgaria
Liliya Sivcheva	Multiprofile Hospital for Active Treatment - Hristo Botev, First Department of Internal Medicine Vratsa, Bulgaria
Jiri Schwarz	Institute of Hematology and Blood Transfusion, Prague, Czech Republic
Jiri Mayer	University Hospital Brno, Clinic of Internal Medicine - Hematology and Oncology, Brno, Czech Republic
Petr Dulicek	University Hospital Hradec Kralove, Department of Clinical Hematology, Hradec Kralove, Czech Republic
Olga Cerna	University Hospital Kralovske Vinohrady, Clinic of Internal Hematology, Prague, Czech Republic
Jean-Jacques Kiladjian	Université de Paris, CIC 1427, Inserm, Paris, France, Centre d'Investigations Cliniques, AP-HP, Hôpital Saint-Louis, Paris, France
Lydia Roy	University of Poitiers, INSERM, CHU Poitiers, Clinical Investigation Centre CIC1402, Department of Onco-Hematology, Poitiers, France
Mathieu Puyade	University of Poitiers, INSERM, CHU Poitiers, Clinical Investigation Centre CIC1402, Department of Onco-Hematology, Poitiers, France
Emilie Cayssials-Caylus	University of Poitiers, INSERM, CHU Poitiers, Clinical Investigation Centre CIC1402, Department of Onco-Hematology, Poitiers, France
Jose Miguel Torregrosa-Diaz	University of Poitiers, INSERM, CHU Poitiers, Clinical Investigation Centre CIC1402, Department of Onco-Hematology, Poitiers, France
Jerome Rey	Paoli-Calmettes Institute, Marseille, France
Dominik Wolf	University Hospital Bonn, Centre for Internal Medicine, Department of Internal Medicine III - Oncology, Hematology and Rheumatology, Bonn, Nordrhein-Westfalen, Germany
Steffen Koschmieder	Department of Hematology, Oncology, Hemostaseology, and Stem Cell Transplantation, Faculty of Medicine, RWTH Aachen University, Aachen, Nordrhein-Westfalen, Germany
Uwe Platzbecker	University Hospital Carl Gustav Carus, Medical Clinic and Polyclinic I, Dresden, Germany
Miklos Egyed	Kaposi Mor Teaching Hospital, Department of Internal Medicine II, Kaposvar, Somogy, Hungary
Tamas Masszi	St Istvan and St Laszlo Hospital of Budapest, Department of Hematology and Stem Cell Transplantation , Budapest, Hungary
Arpad Illes	University of Debrecen, Faculty of Medicine, Department of Hematology, Debrecen, Hungary
Zita Borbenyi	University of Szeged, Albert Szent-Gyorgyi Clinical Centre, Department of Internal Medicine II and Cardiology Centre, Hematology, Szeged, Hungary
Janos Jakucs	Bekes County Pandy Kalman Hospital, 1st Department of Medicine, Gyula, Hungary
Mario Cazzola	Foundation IRCCS Policlinico San Matteo, Pavia, Italy
Jolanta Starzak-Gwozdz	Frederic Chopin Provincial Teaching Hospital No. 1 in Rzeszow, Department of Hematology, Rzeszow, Poland
Krzysztof Warzocha	Institute of Hematology and Transfusion Medicine, Teaching Department of Hematology, Warsaw, Poland
Malgorzata Calbecka	Nicolaus Copernicus Municipal Specialist Hospital, Department of Hematology, Torun, Poland
Maria Soroka-Wojtaszko	Independent Public Teaching Hospital No.1 in Lublin, Department of Hematology-Oncology, Bone Marrow Transplantation and Chemotherapy, Lublin, Poland
Dorota Krochmalczyk	University Hospital in Krakow, Teaching Unit of the Hematology Department, Krakow, Poland
Aleksander Skotnicki	University Hospital in Krakow, Teaching Unit of the Hematology Department, Krakow, Poland
Nicoleta Berbec	Coltea Clinical Hospital, Bucharest, Romania
Horia Bumbea	Bucharest University Emergency Hospital, Bucharest, Romania
Andrei Cucuianu	"Prof. Dr. Ion Chiricuta" Institute of Oncology, Hematology Department, Cluj-Napoca, Romania
Delia Monica Dima	"Prof. Dr. Ion Chiricuta" Institute of Oncology, Hematology Department, Cluj-Napoca, Romania
Emanuil Gheorghita	Rapid Diagnostic Polyclinic SRL. Brasov, Romania
Mihaela Lazaroiu	Rapid Diagnostic Polyclinic SRL, Brasov, Romania
Alexander Myasnikov	Baranov Republican Hospital, Petrozavodsk, Republic of Karelia, Russian Federation
Irina Sokolova	Komi Republican Oncology Centre, Syktyvkar, Russian Federation
Elena Volodicheva	Tula Regional Clinical Hospital, Tula, Russian Federation
Viktor Rossiev	V.D. Seredavin Samara Regional Clinical Hospital Samara, Russian Federation
Vera Yablokova	Yaroslavl Regional Clinical Hospital, Department of Hematology, Yaroslavl, Russian Federation
Anna Vallova	University Hospital with Outpatient Clinic F.D. Roosevelt Banska Bystrica, Banska Bystrica, Slovakia
Antonia Hatalova	University Hospital Saint Cyril and Metod Bratislava, Bratislava, Slovakia
Mikulas Hrubisko	University Hospital Saint Cyril and Metod Bratislava, Bratislava, Slovakia
Carlos Besses Raebel	Hospital del Mar, Department of Hematology, Barcelona, Spain
Halyna Pylypenko	Cherkasy Regional Oncology Centre, Department of Clinical Trials, Cherkasy, Ukraine
Polina Kaplan	Dnipropetrovsk City Multispecialty Clinical Hospital #4, Dnipro, Ukraine
Zvenyslava Masliak	Institute of Blood Pathology and Transfusion Medicine, Department of Hematology, Lviv, Ukraine
Sergiy Klymenko	National Research Centre for Radiation Medicine Institute of Clinical Radiology, Department of Hematology and Transplantation, Kyiv, Ukraine
Tamila Lysa	O.F. Herbachevskyi Regional Clinical Hospital, Hematology Centre, Zhytomyrv, Ukraine

Supplementary results

Patient disposition

Figure S1: CONSORT flow diagram

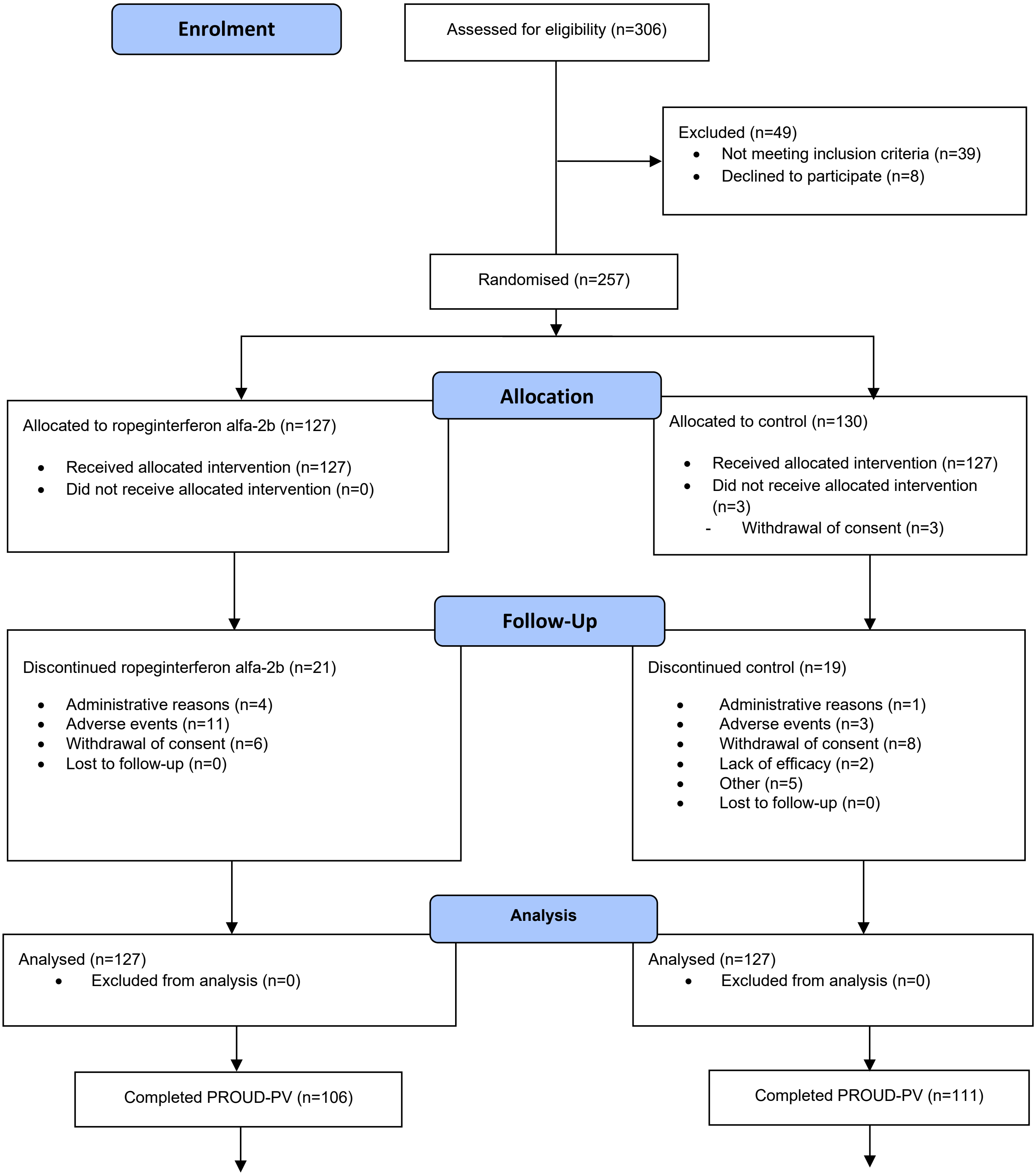

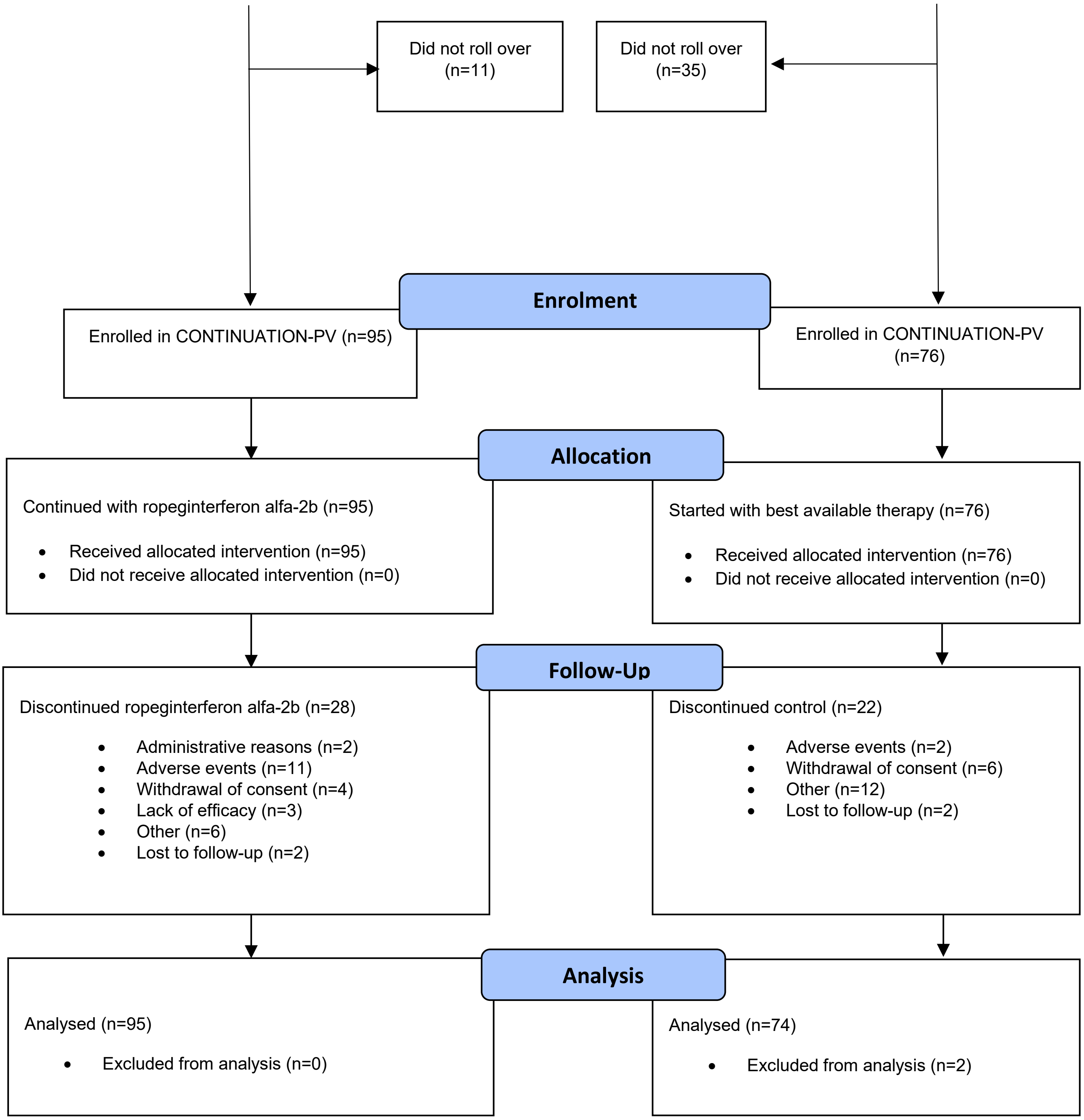

Details of risk events

The first risk events comprised two deaths (due to pneumonia and unspecified causes), one case of myelofibrosis and two thromboembolic events (splenic infarction concurrent with truncus coeliacus thrombosis, and hemorrhagic transformation stroke) in the ropeginterferon alfa-2b arm, and three deaths (due to pneumonia, road traffic accident, and unspecified causes), two cases of myelofibrosis, two cases of acute leukemia and five thrombotic events (two cases of thrombophlebitis, cerebrovascular accident, venous thrombosis of the limb and pulmonary microemboli) in the control arm.

JAK2V617F allele burden

Figure S2: Median *JAK*2V617F allele burden over 72 months (last observation carried forward)

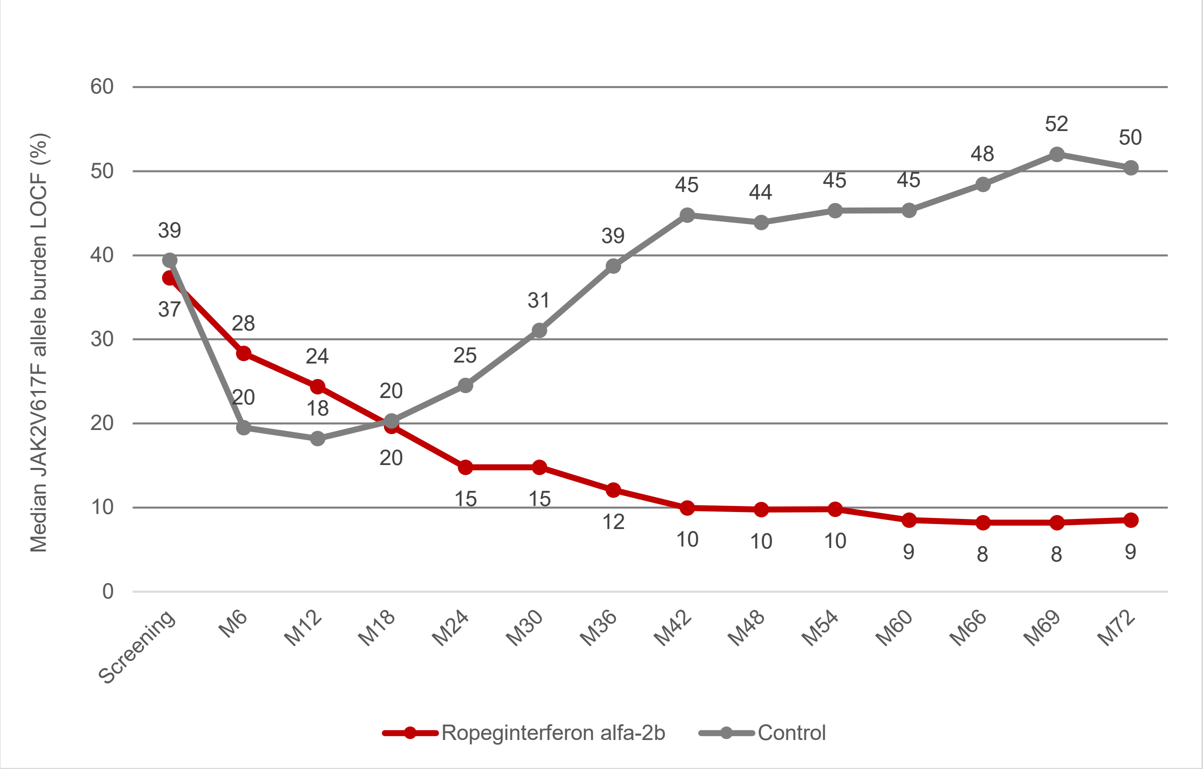

Hematologic response

Figure S3: Complete hematologic response rate over 72 months (last observation carried forward)

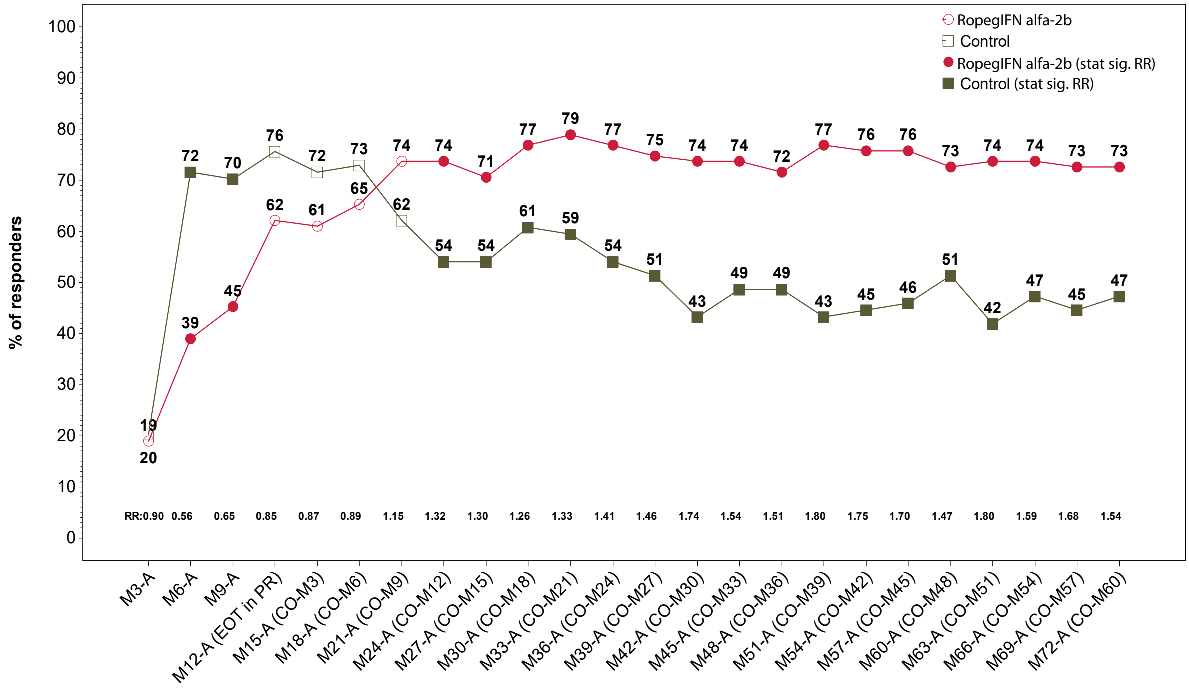

Peripheral blood counts: individual responses over time based on ELN targets

Figure S4: Follow-up time spent in peripheral blood count remission based on European LeukemiaNet response criteria (hematocrit <45% with no phlebotomy in the last 3 months [Panel A]; leukocyte count <10 x 10^9/L [Panel B]; platelet count <400 x 10^9/L [Panel C]) for individual patients with PV (CONTINUATION-PV full analysis set; ropeginterferon alfa-2b arm and control arm, i.e. standard care): Patients are ordered by proportion of time in remission (highest to lowest). Blue bars indicate remission at the latest assessment; yellow bars indicate no remission at the latest assessment.

A. Hematocrit

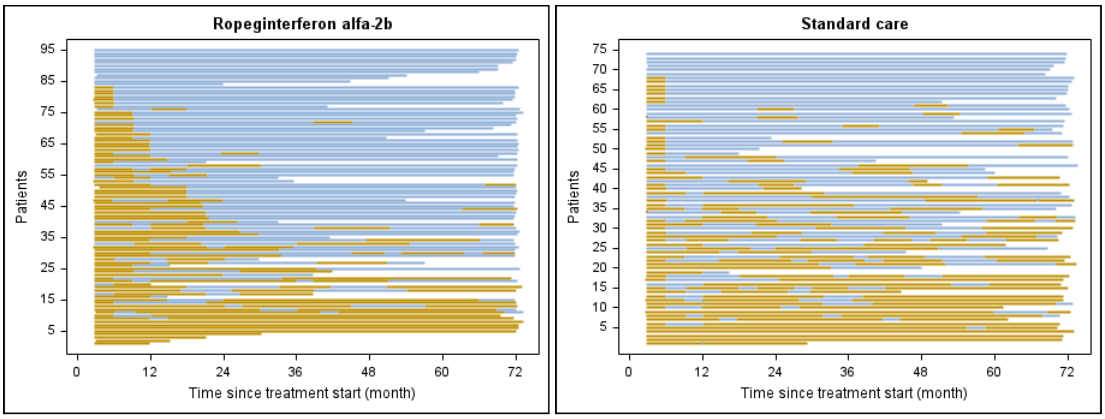

B. Leukocytes

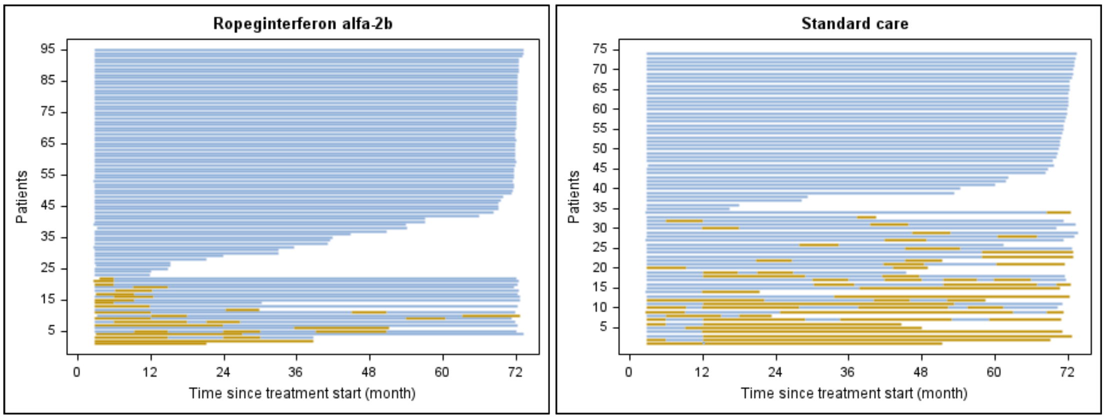

C. Platelets

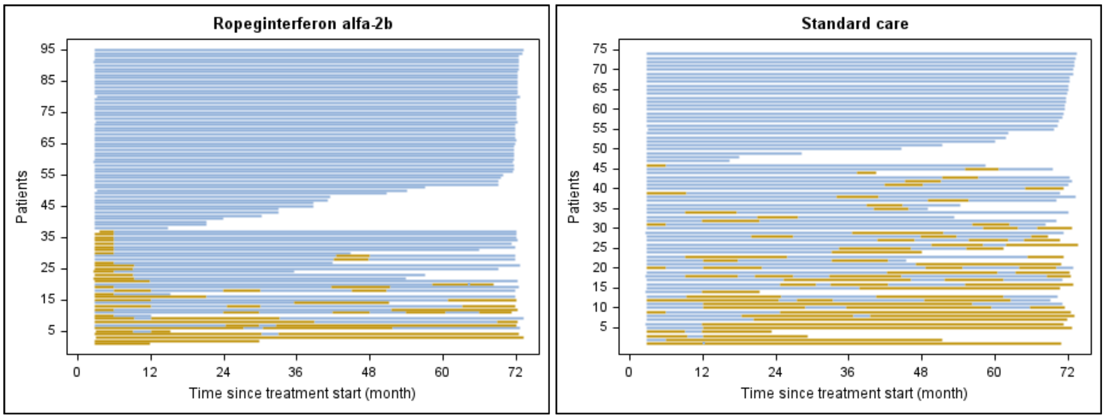

Supplement: Supplementary file 1 — Supplement [file 41375_2023_2008_MOESM1_ESM.pdf]
